# Progress in Disease of Digestive Organs

**Published:** 1902-04-19

**Authors:** 


### Progress in Disease of Digestive Organs.

(*Continued from page* 24.)

**Cirrhosis.**—T. K. Crook[23] found that out of 4,000 fatal cases in America there were 40 not more than 15 years old. Spirits taken at other than meal times were the most important exciting cause. Syphilis, malaria, typhoid, highly seasoned food, mineral poisons also led to it. Hatfield had collected 63 cases in children, alcohol was in these the most frequent single cause and was reported in a sixth of the cases, but syphilis, infectious diseases, and irregular feeding, even an excess of coffee, had been found as exciting causes. G. Converse regards a milk diet as desirable for weeks or months in treatment. An interesting case with regard to the production of biliary or Hanot's cirrhosis is recorded by A. E Thayer.[24] The patient had an undetermined illness, and after three or four years of health was attacked with jaundice and pyrexia, emaciated rapidly and died in a few months. There was complete stenosis of the common duct, proliferation confined to the biliary passages, but no unilobular cirrhosis. The writer asks why it was not present in this case if biliary cirrhosis depends on the passage of a toxin through the biliary passages. There was also interstitial pancreatitis and some of the pancreatic cells were bile-stained. Now it has been shown by E. L. Opie[25] that a gall-stone impacted in the common duct may lead to necrosis of the pancreatic cells and hæmorrhage with inflammatory changes from the entrance of bile into the pancreas. The same results have been obtained in dogs by the injection of bile into the pancreatic duct. R. C. Cabot[26] confirms Courvoisier's law that when the bile duct is obstructed by stone, the gall-bladder does not usually become dilated, but it does when the obstruction is due to other causes; the explanation probably being that in the first case there are usually other stones in the bladder which by their irritation cause contraction. Rupture of the abdominal wall from ascites is very rare, but Merklen and Gougelet[27] record the distension and rupture of the cicatrix of an umbilical hernia. The fluid rapidly drained away, but death from peritonitis followed on the seventh day. Rupture has been recorded elsewhere in the abdominal wall, and even into the scrotum through a hydrocele. Paracentesis should at once be performed if rupture threatens.

**Dysentery.**—Sulphur is warmly recommended by G. E. Richmond ([28]). He orders 20 grains of sublimed sulphur with 5 grains of Dover's powder every four hours. The diarrhœa and pains are greatly relieved at once, and the passage of blood and mucus stopped in two days. Farinaceous diet alone must be given and continued until the diarrhœa has ceased for a week. Chronic cases do not need the Dover's powder and the sulphur may be given only three times a day. He thinks the treatment is more agreeable and gives much better results than ipecacuanha and magnesium sulphate, and suggests its use in tubercular ulceration of the bowels and summer diarrhœa. M. H. Bowman[29] gives a most interesting account of dysentery in the Philippines. There are, he says, three distinct diseases, (*a*) that due to Shiga's bacillus, which never causes ulceration of the colon or liver abscess. The period of incubation here is 48 hours, the onset of the attack is sudden and the patient may die from toxæmia and exhaustion. The bacillus is constantly present, and has produced the disease when given by the mouth, and is agglutinated by the blood of the patients. Amœbic dysentery (*b*) is quite distinct, and causes ulcers of the colon and hepatic abscess, as well as acute typical dysentery, and sometimes recurrent attacks of mild diarrhœa alternating with constipation. Confusion has arisen about amœbic dysentery, because there are two kinds of amœba, the small harmless form and the large pathogenic one. The former may exist in healthy persons, but Strong has shown that the large form if injected into cats always produces typical lesions of the colon. Lastly there is (*c*) simple catarrhal dysentery, with no specific organism, which usually tends to recovery. The writer does not believe in either ipecacuanha or mangnesium sulphate, but in early stages of amœbic dysentery finds the rectal injection of weak quinine solutions efficacious. H. W. Syers[30] reports a fatal case of idiopathic dilatation of the colon in a man aged 59. The condition lasted little over a year; laparotomy was performed at the last moment, and two gallons of fluid drawn off with an enormous quantity of gas, but the patient sank. No previous treatment, except the use of a long rectal tube, had given any relief. Even after death, the transverse colon measured 15 inches around it, and during life the distension was enormous. No tumour or stricture existed, and the bowels were loose throughout; towards the end severe vomiting set in. The solar plexus was atrophic and the intestines showed atrophic catarrh, but no cause of the disease could be discovered. Such cases are extremely rare, and only about 12 are on record.

[23] Med. Rec., Jan. 4. [24] Med. Rec., Jan. 18. [25] Johns Hopkins Bulletin, Nos. 121-3. [26] Med.Rec., Oct. 19. [27] Lancet, Nov. 2. [28] Lancet, Nov. 23. [29] Jour. Tropic. Med., Dec. 16. [30] Lancet, Jan. 4.

